# Association of IL-17F rs763780 polymorphism and risk of psoriasis in Turkish population: a case-control study^☆^

**DOI:** 10.1016/j.abd.2023.06.006

**Published:** 2024-02-07

**Authors:** Ayşegül Başak Akadam-Teker, Burak Akşan

**Affiliations:** a Department of Medical Genetics, Giresun University, Faculty of Medicine, Giresun, Turkey; b Department of Skin Diseases, Giresun University, Faculty of Medicine, Giresun, Turkey

**Keywords:** Cytokines, İnterleukin-17F, Polymorphism, Psoriasis

## Abstract

**Background:**

Psoriasis represents a chronic inflammatory phenotype shaped by genetic interactions, characterized by keratinocyte hyperproliferation and commonly affecting the skin and joints. Experimental and clinical studies suggest that the IL-17F gene locus plays a role as a central cytokine in the immunopathogenesis of psoriasis.

**Objectives:**

Based on the central role of IL-17F in the pathogenesis of psoriasis, the authors thought that variations in this gene could affect the susceptibility and severity of this disease. Therefore, in this study, the authors aimed to analyze whether the IL-17F rs763780 variant has an effect on psoriasis pathogenesis in the Turkish population.

**Method:**

In this case-control study, the study group consisted of 603 people (201 psoriasis patients (73 males/128 females)/402 controls (146 males/256 females) were genotyped in terms of IL-17F rs763780 polymorphism with TaqMan 5' Allelic Discrimination Test.

**Results:**

The genotype distributions of the IL-17F rs763780 polymorphism between patients and control groups were statistically different, and the TC (heterozygous genotype) and CC (homozygous mutant genotype) genotypes were more represented in the patients group than in the control group (24.9% vs. 10.2%; 2.0% vs. 0.2%, respectively). In addition, the variant C allele was higher in the patients group and this was statistically significant (p < 0.001), and the C allele carriage was associated with a 3.14-fold increased risk of psoriasis (95% CI 2.015‒24.921).

**Study limitations:**

The present study has some limitations. The first limitation is the relatively small sample size. The second limitation is that the authors could not measure IL-17F expression levels. However, the present study data draw attention to the importance of IL-17F which deserves to be studied in a larger sample group.

**Conclusion:**

We report that IL-17F rs763780 TC and CC genotype and C allele are associated with an increased risk of psoriasis in the Turkish population.

## Introduction

Psoriasis represents a chronic inflammatory phenotype shaped by genetic interactions, characterized by keratinocyte hyperproliferation and commonly affecting the skin and joints.^1^ It affects 1%‒3% of the world's population and individuals who were suffering from this disease, also have to cope with the psychological and physiological problems caused by the disease.^2^, ^3^ It is very important to identify individuals at risk for psoriasis with significant comorbidities, including cardiovascular disease, diabetes and depression, and to review early treatment options. Although the complex etiology mediated by immune mediators has not been fully elucidated yet, the discovery of the interleukin-23/interleukin-17 axis which is important in the pathophysiology of psoriasis has changed this perspective on the treatment options of the disease and has drawn attention to the IL-17 family, one of the important mediators in this pathway.^4^, ^5^, ^6^ IL-17A and IL-17F, which are members of the IL-17 family and have approximately 50% structural homology, play a central role in the pathogenesis of psoriasis and both have been associated with immune-mediated inflammatory diseases, including psoriasis.^7^, ^8^, ^9^, ^10^ Both IL-17A and IL-17F work to upregulate the inflammatory response by showing complex interactions with proinflammatory cytokines.^11^ Reporting of increased levels of both IL-17A and IL-17F mRNA in synovial fluid from psoriatic arthritis patients supports this dual association.^12^ IL-17A blocking agents are widely accepted today in the treatment of psoriasis but because of the difference in individual responses to treatment and loss of response over time have led to the formation of alternative treatment options like Bimekizumab has emerged as a new strategy which is targeting both IL-17A and IL-17F as a new agent. Based on clinical and experimental study data on this new treatment strategy, the authors can say that dual blockade provides improvement in disease symptoms in a short time and a better anti-inflammatory response is obtained in the regulation of inflammatory process-related genes compared to treatments targeting only IL-17A.^12^, ^13^, ^14^, ^15^ As a result of evaluating this information, it seems possible to say that the IL-17F gene locus plays a role as a central cytokine in the immunopathogenesis of psoriasis. In addition, demonstrating the increased production of IL-6 and IL-8 by IL-17F induction in the lesional skin of psoriasis patients has once again underlined the role of IL-17F in psoriasis pathogenesis.^16^, ^17^ Finally, the report that IL-17F knockout mice were associated with higher resistance to psoriasis compared to IL-17A knockout mice indicates that IL-17F is closely related to psoriasis.^18^ Despite its central role in the pathogenesis of psoriasis, the number of studies investigating the effect of variations in the IL-7F gene locus on the pathogenesis of the disease is quite limited and moreover, there is no data related to this issue in Turkish society. Genetic components can affect not only disease susceptibility, but also clinical type, age of onset, severity, and even risk of psoriatic arthritis. Therefore, the authors aimed to investigate the possible relationship of the IL-17F rs763780 variant with the disease.

## Methods

### Study Design

The present study protocol was approved by the Ethics Committee of the Giresun University Faculty of Medicine (ethical committee number: KAEK-115) and written informed consents were obtained from all patients. All procedures performed in this study were in compliance with the ethical standards of the institutional and/or national research committee and the Declaration of Helsinki World Medical Association.^19^ In this study, 201 psoriasis patients who applied to Giresun University A. İlhan Özdemir Training and Research Hospital between 2019‒2021, diagnosed clinically or histopathologically, were included in the present study group. As the control group, 402 individuals who applied to the dermatology outpatient clinic with a dermatological disease and were age-matched (with no clinical evidence of psoriasis or other autoimmune disorders ) were included in the study group. These individuals had no history of psoriasis or any chronic inflammatory disease. The demographic and clinical characteristics of both psoriasis patients and the control group were recorded in synchronized Excel files for later use. Psoriatic arthritis was investigated in all patients. Children (age < 18) (n = 8), pregnant and lactating participants (n = 3), and participants (n = 13) with a history of any immunological or inflammatory disease were excluded from the study.

### Interleukin-17F rs763780 genotype analysis

DNA isolations were made with a commercial kit (Roche high pure isolation kit, Germany) from the peripheral blood taken from patients in the study, and the DNA levels were calculated by determining the purity and stored at +4 °C until the time of the study. Allelic variations IL-17F rs763780 were genotyped with the Real-Time PCR method and bi-directional quantitative TaqMan 5' Allelic Discrimination Test (Applied Biosystems, Foster City, CA) using established protocols according to the manufacturer's instructions. The PCR amplification conditions were performed at 95 °C for 10 minutes, at 95 °C for 15 seconds, 55 °C for 1 minute, and 72 °C for 1 minute 45 cycles For the control, 10% of randomly selected samples were double genotyped and the accuracy of the results was confirmed again.

### Statistical analysis

The statistical analysis of this study was made using the SPSS 20 package program. Statistical significance was taken as p < 0.05. Alleles and genotype frequencies were calculated by direct counting. Genotype distributions in study groups were analyzed with Hardy-Weinberg Equilibrium (HWE) compatibility. The Chi-Square (χ^2^) test was used to evaluate the intergroup differences in the frequency of genotype and alleles. Odds Ratio (OR) and 95% Confidence Interval (95% CI) are given to determine the risk factor between groups. In order to determine the relationship between psoriasis risk and IL-17F rs763780 C allele carriage, a binary logistic regression model and multivariate regression analysis were performed with a significance of p < 0.05. Psoriasis was used as a dependent variable.

## Results

The information on the demographic characteristics of the working groups is in Table 1. Psoriatic arthritis was observed in 46 of the psoriasis patients, no signs of psoriatic arthritis were found in 155 patients. When the authors divided the present patients into two groups according to the age of onset of psoriasis, the number of early-onset (age ≤ 40) psoriasis patients was 132 and the number of late-onset (age > 40) psoriasis patients was 69. In 35 of the patients, there was a history of psoriasis in first-degree relatives, and in 166 patients no history of psoriasis was identified in first-degree relatives. The number of patients whose Psoriasis Area and Severity Index (PASI) score was ≤10 was 28, and the rest of the psoriasis group, 173 patients had PASI score of >10. Genotype distributions and allele fractions of study populations are given in Table 2. Both patient and control groups showed compatibility with Hardy-Weinberg Equilibrium (HWE). There is a statistically significant difference between the patient and control groups in terms of genotype distributions (p < 0.001). TC and CC genotypes were more represented in the patient group than in the control group (24.9% vs. 10.2%; 2.0% vs. 0.2%, respectively). In addition, the variant C allele was higher in the patient group and this was statistically significant (p < 0.001), and the C allele carriage was associated with a 3.14-fold increased risk of psoriasis (95% CI 2.015‒24.921). When the genotype distributions in the patient group were examined in terms of family history, psoriatic arthritis, age of onset, and gender, no statistical difference was observed (data not shown). The results of the logistic regression analysis to prove the relationship between psoriasis and the IL-17F C allele also confirmed that the variant C allele increases the risk of psoriasis (p < 0.001; 95% CI 2.015‒4.491).

**Table 1:** Demographical characteristics of the study population.

		Cases (n = 201)	Controls (n = 402)	p-value
**Age (years) (mean ± SD)**		44.08 ± 14.17	47.61 ± 12.0	NS
**Sex, n (%)**				NS
Male		73 (36.3%)	146 (36.3%)	
Female		128(63.7%)	256 (63.7%)	
**Arthritis**				
Arthritis+		46 (22.5%)	‒	
Arthritis-		155(77.5%)		
**Age of onset, n (%)**	≤ 40	132 (66.0%)	‒	
	> 40	69 (34.0%)		
**Family History (FH), n (%)**	FH+	35 (17.0%)	‒	
	FH-	166 (83.0%)		
**PASI**	> 10	173 (86.0%)	‒	
	≤ 10	28 (14.0%)		

Mean values were compared between patients and controls by using the Student's *t*-test. Qualitative data were analyzed by the Chi-Square test. NS, Not Significant, PASI, Psoriasis Area and Severity Index. Data are presented as mean ± S.D and n (%). Bold values were statistically significant (p < 0.05). n:number of samples.

**Table 2:** Genotypes and allele fraction in study groups.

Genotip	Cases (n = 201)	Controls (n = 402)	χ^2^	p-value
TT	147 (73.1%)	360 (89.6%)	28.322	**< 0.001**
TC	50 (24.9%)	41 (10.2%)		
CC	4 (2.0%)	1 (0.2%)		
HWE	p < 0.05	p < 0.05		
T allele	0.98	0.99	3.050	0.081
C allele	0.26	0.10	26.983	< **0.001**

Genotypes and allele fractiones were analyzed by the Chi-Square test. Data are presented as n (%). Bold values were statistically significant (p < 0.05). n, Number of samples; HWE, Hardy-Weinberg Equilibrium.

## Discussion

There are 6 members of the IL-17 family (IL17A-IL17F), which are determined to play a critical role in psoriasis pathogenesis.^20^, ^21^, ^22^ The most studied of these family members are IL-17A and IL-17F, which have similar biological functions by showing 50% homology. Both of them have been frequently associated with many inflammatory diseases, including psoriasis.^23^, ^24^, ^25^ Although IL-17A appears to play a central role in the pathogenesis of psoriasis, recent studies have reported an increasing level of IL-17F in lesional skin in psoriasis, in addition to the demonstration that IL-17F has similar functions as IL-17A and IL-17F has begun to focus on its possible critical role in the immunopathogenesis of the disease.^26^ Recent study results by Bertelsen et al. provided evidence that IL-17F plays a key role in the IL-23/IL-17 axis^26^ have shown that IL-17F stimulation induces the expression of IκBκ, whose critical role in psoriasis pathogenesis has been previously demonstrated^27^ in human keratinocytes by changing both mRNA and protein levels, and plays a role in the regulation of many genes such as psoriasis associated.^26^ Other accumulating evidence has reported that the IL-17F rs763780 variant produces different drug responses to administered therapeutic approaches in individuals. This situation has once again highlighted the critical role of IL-17F in psoriasis pathogenesis.^28^ The researchers evaluated the PASI 75 response in a study group of 194 psoriasis patients and associated the IL17F rs763780 TT genotype with a better response to treatment with ustekinumab. Data from another study in which ustekinumab was administered also reported a decrease in IL-17A and IL-17F levels at week 24, but this decrease was significant only between IL-17F and placebos.^29^ Although the role of IL-17F in psoriasis immunopathogenesis has not been fully clarified, determining the variants on this gene has great importance in terms of creating patient-specific treatment options. However, there are a few studies on this subject in the literature and this situation is quite limited. One of these studies Kaur et al. associated the IL-17F rs763780 variant with increased psoriasis susceptibility in their studies.^30^ The present study observed that genotypes carrying the variant allele were higher in the patient group compared to the control group which is similar to the data of this study. In addition, the authors would like to report the finding that the variant allele is associated with a 3.1-fold increased risk of psoriasis. Similarly, Choi et al., who defined IL-17F as a new risk locus for psoriasis, reported that genotypes carrying the variant allele were more widely represented in the patient group.^31^ Consistent with this, another study from the Korean population reported that the homozygous variant genotype was associated with a 3.27-fold increased risk of psoriasis.^32^ The data of studies conducted in Japanese and Spanish populations are also inconsistent with these studies.^33^ studies reported no association with the IL-17F rs763780 variant with psoriasis. This can be explained by the fact that a gene is dependent on its genomic context and may exhibit different expression patterns in different ethnic groups.^34^ As a result; based on the information the authors obtained from the present data, we would like to underline the relationship of IL-17F with psoriasis. In addition, the authors had the impression that IL-17F rs763780 variant induced disease susceptibility by the C allele. Since psoriasis has high comorbidities, it is important to determine the risky groups and to apply early treatment options. It can be recommended as an appropriate biomarker for clinicians as appropriate treatments by determining the correct target will lead to a decrease in the morbidity of the disease.

### Limitations

However, this study has some limitations. The first limitation is the relatively small sample size. A larger sample size will increase statistical power. The second limitation is that the authors could not measure IL-17F expression levels. However, the present study data draw attention to the importance of IL-17F and the study deserves to be studied in a larger sample group.

## Conclusion

Based on the information the authors obtained from the present data, we would like to underline the relationship of IL-17F with psoriasis. In addition, IL-17F rs763780 TC and CC genotype and C allele are associated with an increased risk of psoriasis in the Turkish population, according to this sample. Since psoriasis has high comorbidities, it is important to determine the risky groups and to apply early treatment options. It can be recommended as an appropriate biomarker for clinicians as appropriate treatments by determining the correct target will lead to a decrease in the morbidity of the disease.

## Financial support

The present study was supported by a grant from Scientific Reserch Coordination Unit of Giresun University (Project n° SAĞ-BAP-A-250620-66).

## Authors’ contributions

Ayşegül Başak Akadam-Teker: Design the Research, supply samples, analyze the data, literature search, draft the article, and perform the laboratory work.

Burak Akşan: Supply samples, analyze the data, and literature search.

## Conflicts of interest

None declared.
